# Hypoxia-inducible factor prolyl hydroxylase inhibitor roxadustat (FG-4592) protects against renal ischemia/reperfusion injury by inhibiting inflammation

**DOI:** 10.1080/0886022X.2021.1915801

**Published:** 2021-05-10

**Authors:** A-Feng Miao, Jian-Xiang Liang, Lei Yao, Jun-Ling Han, Li-Juan Zhou

**Affiliations:** a Department of Nephrology, Taizhou People’s Hospital, Fifth Affiliated Hospital to Nantong University, Taizhou, Jiangsu, China; b Department of Ultrasonography, Weifang People's Hospital, Weifang, Shandong, China; c Department of Anesthesiology, Second Affiliated Hospital of Nantong University, Nantong, Jiangsu, China; d Clinical Laboratory, Taizhou People’s Hospital, Fifth Affiliated Hospital to Nantong University, Taizhou, Jiangsu, China

**Keywords:** FG-4592, renal ischemia/reperfusion injury, inflammation

## Abstract

Hypoxia-induced inflammation is the critical pathological feature of acute kidney injury (AKI). Activation of hypoxia-inducible factor (HIF) signaling is considered as a central mechanism of body adapting to hypoxia. Hypoxia-inducible factor prolyl hydroxylase inhibitor FG-4592 (Roxadustat) is a first-in-class HIF stabilizer for the treatment of patients with renal anemia. The current study aimed to investigate whether FG-4592 could protect against ischemia/reperfusion (I/R)-induced kidney injury *via* inhibiting inflammation. Here, efficacy of FG-4592 was evaluated in a mice model of I/R-induced AKI. Interestingly, improved renal function and renal tubular injuries, combined with reduced kidney injury molecule-1 were observed in the mice with FG-4592 administration. Meanwhile, inflammation responses in FG-4592-treated mice were also strikingly attenuated, as evidenced by the decreased infiltration of macrophages and neutrophils and down-regulated expression of inflammatory cytokines. *In vitro*, FG-4592 treatment significantly protected the tubular epithelial cells against hypoxia-induced injury, with suppressed inflammation and cell injuries. In summary, FG-4592 treatment could protect against the I/R-induced kidney injury possibly through diminishing tubular cells injuries and suppression of sequence inflammatory responses. Thus, our findings definitely offered a clinical potential approach in treating AKI.

## Introduction

As a multifaceted syndrome, acute kidney injury (AKI) occurs in approximately 15% of patients admitted to hospital, affecting more than 13 million people per year worldwide [1]. Ischemic injury has long been the major causes of AKI. Unfortunately, despite improvements in AKI management, there is no exact therapy to treat AKI [2]. Accumulating evidence indicated that acute inflammatory responses is one of the critical pathological features of AKI, characterized by infiltration of inflammatory cells (macrophage and neutrophils) and release of inflammatory cytokines [3]. Moreover, targeting inflammation is an important strategy for treating AKI [4].

The decline in oxygen tension, a direct consequence of ischemic injury, could rapidly lead to energy deprivation and thereby contribute to cellular injuries [5]. In fact, hypoxia is identified as a major pathophysiologic mechanism in AKI. Recently, a central mechanism of adaptation to low oxygen tensions operates through activation of the hypoxia-inducible factor (HIF) signaling [6]. HIF could induce endogenous defense against hypoxic injury by activating a widespread range of hypoxia inducible gene expression [7]. HIFs are heterodimers that are composed of an oxygen-dependent α-subunit (HIF-α) and constitutive β-subunit (HIF-β), which are regulated by an enzyme family of HIF-prolyl hydroxylases (PHD).

FG-4592 is a specific small-molecule HIF-PHD inhibitor (HIF-PHI) that is undergoing phase IV trials for the treatment of renal anemia by stimulating HIF activation [8]. Giving that renal HIF system exerts a central role in endogenous defense mechanisms against injury, we speculated that FG-4592 could ameliorate AKI. Moreover, whether FG-4592 can protect against kidney injury *via* inhibiting inflammation remains unclear. Therefore, in the present study, we studied the effects of FG-4592 on ischemia/reperfusion (I/R)-induced kidney injury, as well as the potential mechanisms.

## Material and methods

### Animals

Male C57BL/6 mice (weight 20–25 g) were used. All mice were housed in a standard environment with a 12-h light/dark cycle. All experiments procedures were approved by the ethics review committee for animal experimentation of Nantong University.

### Animal models and therapeutic experiments

#### Ischemia/reperfusion injury

The model of renal-I/R injury (I/RI) was established in mice by clipping the bilateral renal pedicles for 30 min using microaneurysm clamps as previously described [9].

#### HIF-PHI FG-4592 preconditioning

The HIF-PHI FG-4592 was purchased from Selleck Chemicals (Houston, TX, USA). It was dissolved at 50 mg/ml, and then diluted in sterile PBS to 1 mg/ml. The mice in FG-4592 + I/RI group were administrated with FG-4592 for 48 h at a dose of 10 mg/kg/day *via* i.p. injection before I/RI. The sham or I/RI mice received an i.p. injection of vehicle (NC group) [10]. To clarify the effect of FG-4592 on normal kidney, another experiment was performed by the application of FG-4592 or vehicle to the normal mice for 3 days (*n* = 6). Mice were killed at 2 days after I/RI.

### The tubular epithelial cell isolation

Kidneys were harvested from FG-4592 or vehicle-treated mice, and the tubular epithelial cells (TECs) were isolated as previously described [11]. Briefly, the renal cortex was cut into 2-mm^3^-thick fragments. The kidney tissues were ground with a 100-mesh stainless steel sieve, followed by filtering through 150-mesh steel sieves. TECs on the 150-mesh steel sieve were harvested.

### Cell culture and treatment

The HK-2 cells were purchased from ATCC and cultured in DMEM–Ham’s F-12 medium (Gibco) supplemented with 10% fetal bovine serum (Gibco), penicillin (100 IU/ml), and streptomycin (100 μg/ml; Invitrogen). The HK-2 cells were cultured in glucose- and serum-free culture medium under hypoxic conditions (1% O_2_) with a hypoxic chamber (Thermo Scientific, Waltham, MA) for 18 h to induce hypoxic injury. Then, the HK-2 cells were transferred back to normal oxygen conditions (21% O_2_) with regular culture medium for 6 h, serving as an ischemia/reoxygenation (H/R) model. The HK-2 cells in FG-4592-treated group were pretreated with FG-4592 (15 μM) for 6 h before H/R [10].

### Western blotting

Western blotting was performed as previously described [12]. Briefly, total protein from kidney tissue and cells were extracted by lysis buffer (Thermo Scientific, Waltham, MA). Protein concentrations were detected using the BCA Protein Assay Kit (Beyotime Biotechnology, Shanghai, China). Individual immunoblots were probed with primary antibodies as followed: Anti-GAPDH (CW0100M; Cwbio, Beijing, China), anti-KIM-1 (ab190696, Abcam), anti-p-p65 (#3033, CST), anti-Bax (#2772, CST), and anti-cleaved caspase-3 (#9661, CST).

### Kidney morphology and tubular injury scoring

For histology analysis, kidney sections were stained with Periodic Acid-Schiff (PAS). To blindly evaluate tubular injury, 50 images per animal were assessed on PAS-stained sections by a nephropathologist, and semi-quantitatively scored as previously described [13].

### Quantitative real-time PCR assay

The total RNA was extracted using the RNA Extraction Reagent (YIFEIXUE BIO TECH, China), and cDNA was synthesized using PrimeScript RT Reagent Kit (Vazyme, China). The PCR was performed using a 7500 real-time PCR System (Applied Biosystems). Relative expression was normalized to GAPDH levels. All the primers for real-time PCR were listed in the Table 1.

**Table 1.** Sequences of the primers for quantitative real-time PCR.

Gene	Primer sequence (5’–3’)
Mus MCP-1	F: GCTCTCTCTTCCTCCACCAC
	R: ACAGCTTCTTTGGGACACCT
Mus IL-1β	F: ACTGTGAAATGCCACCTTTTG
	R: TGTTGATGTGCTGCTGTGAG
Mus TNF-α	F: TCCCCAAAGGGATGAGAAG
	R: CACTTGGTGGTTTGCTACGA
Mus KIM-1	F: ACATATCGTGGAATCACAACGAC
	R: ACTGCTCTTCTGATAGGTGACA
Mus GAPDH	F: GCATGGCCTTCCGTGTTC
	R: GATGTCATCATACTTGGCAGGTTT
Homo MCP-1	F: CTTGGGTTGTGGAGTGAGTGT
	R: AGCAGAAGTGGGTTCAGGATT
Homo IL-1β	F: GTGGTGGTCGGAGATTCGTAG
	R: GAAATGATGGCTTATTACAGTGGC
Homo TNF-α	F: CGAAGTGGTGGTCTTGTTGCT
	R: CCCGACTATCTCGACTTTGCC
Homo KIM-1	F: CGTCCACCGCAAATGCTT
	R: TCTGCGCAAGTTAGGTTTTGTC
Homo GAPDH	F: GGCATCCACTGTGGTCATGAG
	R: TGCACCACCAACTGCTTAGC

### Immunostaining staining

Immunostaining staining was performed as previously described [11]. Antibodies used were as follows: F4/80 (ab6640, Abcam), neutrophil (ab2557, Abcam), and NF-κB p-p65 (#3033, CST). Immunostained samples were visualized on an Olympus FV-1000 confocal microscope (Olympus, Tokyo, Japan) or a NIKON E100 optical microscope (Tokyo, Japan).

### Statistical analysis

Data are presented as mean ± SD. For comparing two groups, the *t*-test was employed using SPSS (IBM Corp., Armonk, NY). When more than two groups were compared, One-way analysis of variance followed by Bonferroni correction was employed to analyze the differences. A two-sided *p* value < .05 was considered significant difference.

## Results

### Fg-4592 protected against I/R-induced AKI in mice

**Figure 1.**
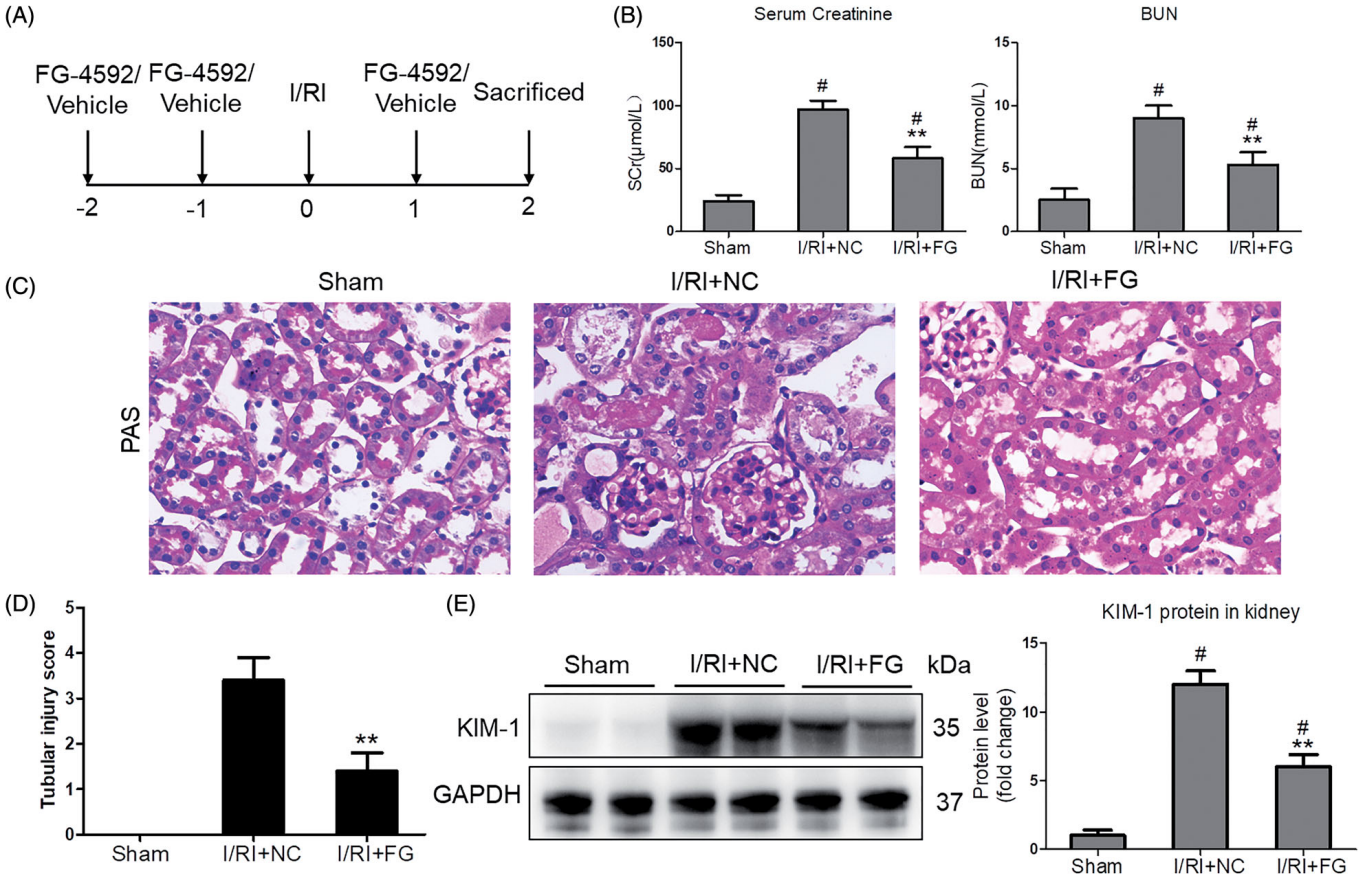
FG-4592 attenuated I/R-induced AKI. (A) Schematic diagram of the experimental design. In brief, mice were administrated with FG-4592 or vehicle before performing ischemia/reperfusion injury. And the mice were sacrificed at 48 h after disease induction. (B) Serum creatinine and BUN levels. (C) Representative images of PAS staining of kidneys (×400). (D) Tubular injury score in mice. (E) Western blot analysis of KIM-1 in renal cortex tissue lysates. *n* = 6 mice per group. Data are presented as mean ± SD, **p < .01 vs. I/RI + NC mice, #*p* < .05 vs. sham mice, ANOVA followed by Bonferroni correction.

Firstly, the effects of FG-4592 on renal function in I/R-induced AKI were explored. The schematic illustration of protocol was shown in Figure 1(A). Interestingly, Scr and BUN levels were significantly decreased after FG-4592 administration (Figure 1(B)). As detected by PAS staining, the tubular injuries, characterized by the dilation of renal tubules, tubular cell necrosis, and appearance of protein casts, were significantly attenuated in mice-administered with FG-4592 (Figure 1(C)). Indeed, decreased tubular injury scores was considerably exhibited in mice with FG-4592 administration (Figure 1(D)). It must be noted that, consistent with previous studies, we did not find side effects of FG-4592 on renal function in mice (Supplementary Figure 1). Furthermore, to clarify the protective role of FG-4592 in I/R-induced renal tubule injury, the tubular injury marker of KIM-1 in the kidneys was examined. Interestingly, the expression of KIM-1 in mice with FG-4592 pretreatment remarkably decreased (Figure 1(E)), indicating that FG-4592 administration obviously attenuated I/R-induced renal dysfunction and tubules injuries.

### Fg-4592 ameliorated the inflammation in I/R-induced AKI

**Figure 2.**
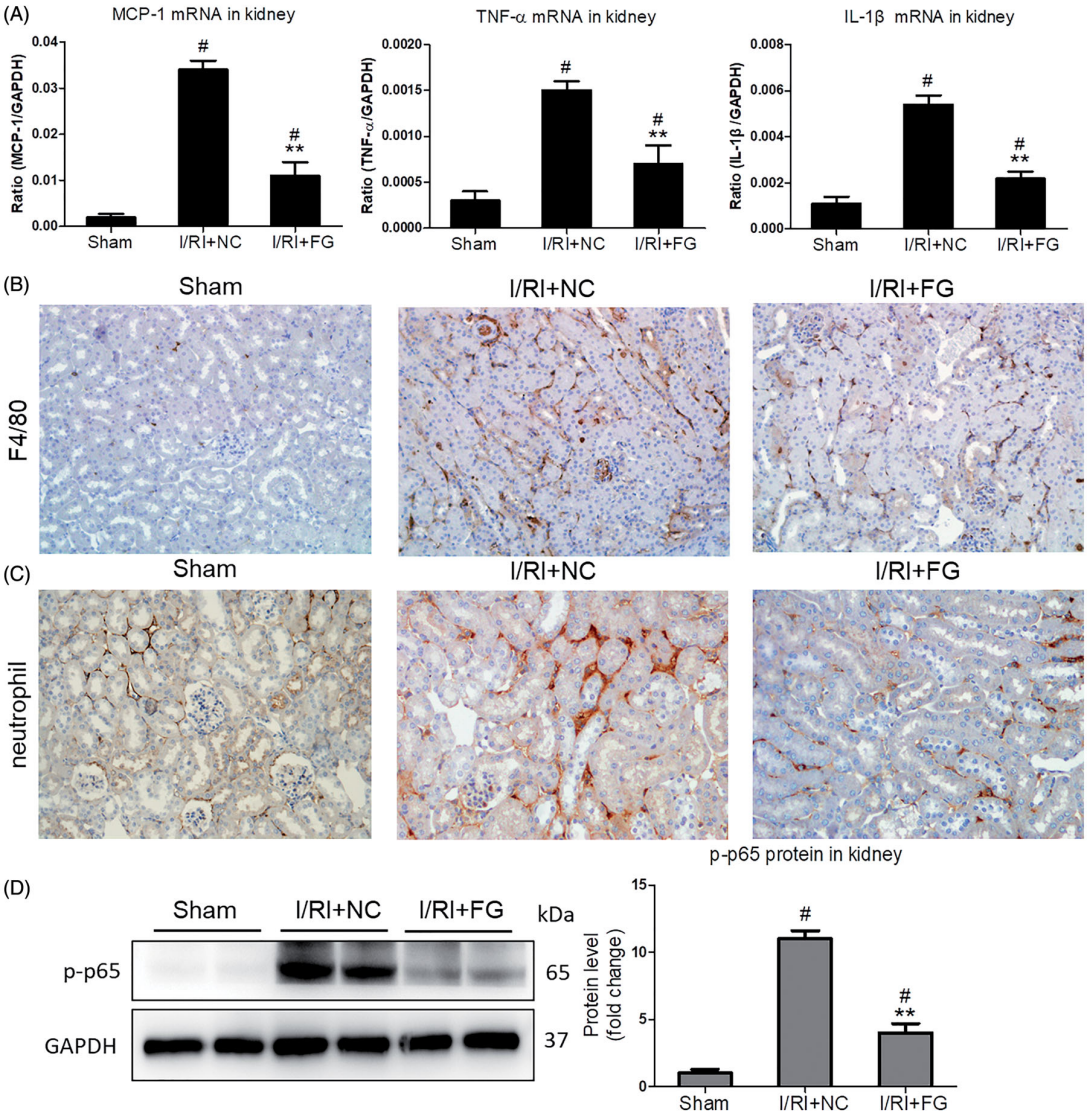
Effects of FG-4592 on renal inflammation in mice challenged with I/R-induced injury. (A) qRT-PCR analysis of cytokine (TNF-α, IL-1β and CCL-2) mRNA expression levels in renal cortex tissue lysates. (B, C) Immunostaining of F4/80 and neutrophils in the kidneys (×200). (D) Western blot analysis of p-p65 in renal cortex tissue lysates of mice (left) and quantitative analysis of the Western blots of p-p65 (right). *n* = 6 mice per group. Data are presented as mean ± SD, **p < .01 vs. I/RI + NC mice, #*p* < .05 vs. sham mice, ANOVA followed by Bonferroni correction.

Accumulating evidence indicated that inflammation played a decisive role in the pathogenesis of AKI. Thus, the effect of FG-4592 on inflammation in I/R-induced AKI was studied. As shown in Figure 2(A), the inflammatory markers including MCP-1, TNF-α, and IL-1β were all markedly ameliorated in the kidneys of mice with FG-4592 administration. Meanwhile, the immunostaining staining showed that FG-4592 markedly inhibited the interstitial infiltration of inflammatory cells (macrophages and neutrophils) (Figure 2(B,C)). Moreover, expression of NF-κB p-p65, the key factor regulating the inflammatory responses, was strikingly decreased in kidneys of mice receiving FG-4592. (Figure 2(D)). These data indicated that FG-4592 protected against I/R-induced AKI by inhibiting inflammation.

### Fg-4592 protected against H/R-induced renal tubular cell injury *in vitro*

**Figure 3.**
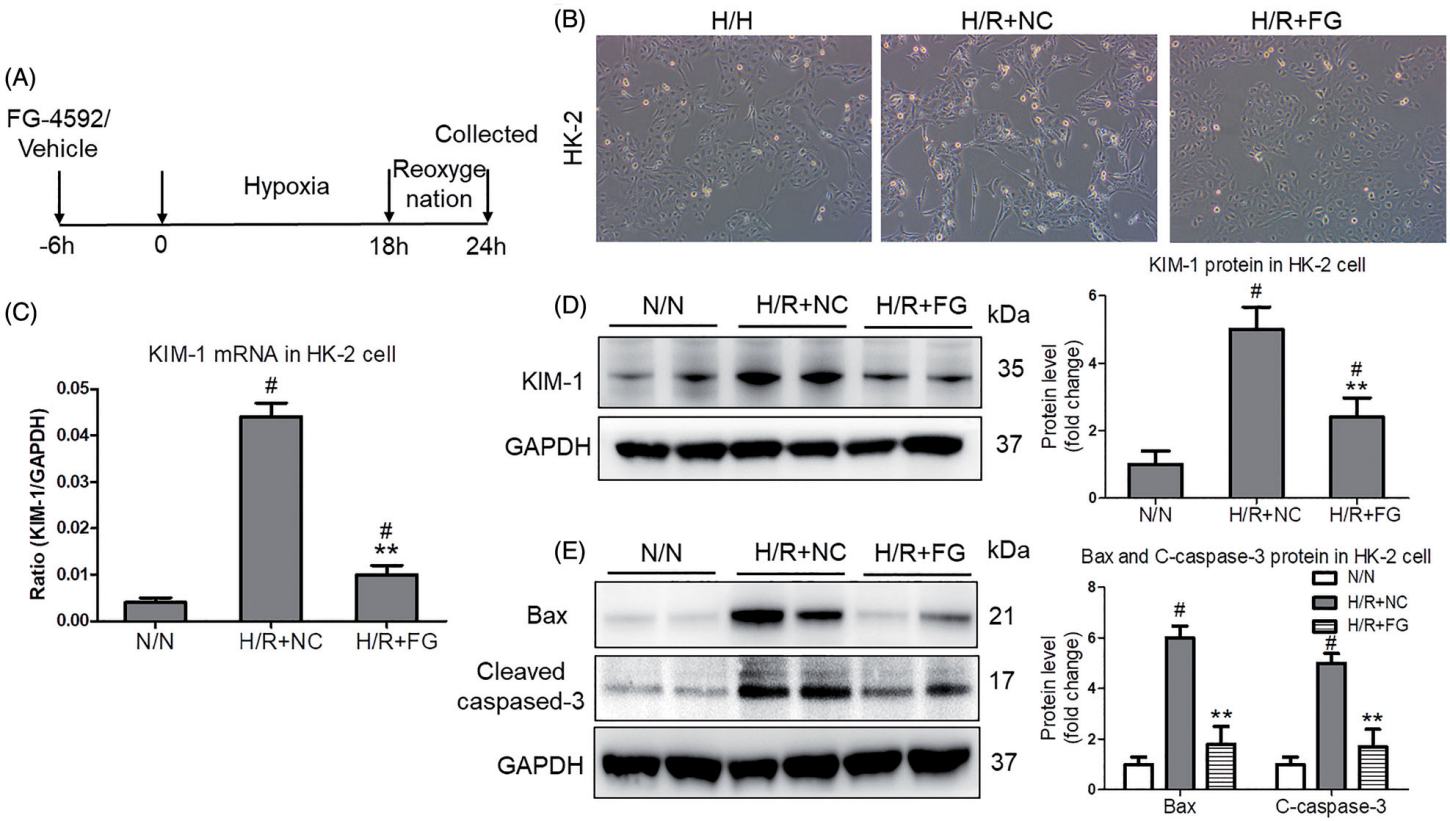
FG-4592 protected against hypoxia-induced HK-2 cells injury. (A) Schematic diagram of the experimental design. In brief, the HK-2 cells were administrated with FG-4592 or vehicle before performing hypoxia/reoxygenation. And the cells were harvested at 6 h after reoxygenation. (B) Representative images of HK-2 cells (×200); (C) qRT-PCR analyses of KIM-1 mRNA expression in HK-2 cells. GAPDH was used as an internal control. (D) Western blotting analysis of KIM-1 protein in HK-2 cells. Quantitative analysis of the Western blots of KIM-1. (E) Western blot analysis of Bax and Cleaved-caspase-3 in HK-2 cells. Quantitative analysis of the Western blots of Bax and C-caspase-3. HK-2 were pretreated with or without FG-4592, then cultured with H/R. GAPDH was used as the loading control. Data were expressed as means ± SD. All experiments were duplicated for three time. **p < .01 vs. H/R + NC group, #*p* < .05 vs. N/N group, ANOVA followed by Bonferroni correction. qRT-PCR: quantitative real-time PCR.

Next, to evaluate the direct effect of FG-4592 on H/R-induced renal tubular cell injury, the HK-2 cells were used. As shown in Figure 3(A), the schematic illustration of experimental protocol was presented. More interestingly, the morphology of HK-2 injury was obviously changed after FG-4592 administration (Figure 3(B)). Moreover, the injury of HK-2 cells induced by hypoxia significantly ameliorated when pretreated with FG-4592 at 15 μM, as evidenced by the results of KIM-1 mRNA and protein expression, which were detected by qRT-PCR and western blotting, respectively (Figure 3(C,D)). Indeed, the expression of Bax and cleaved-caspase-3 proteins was also decreased after FG-4592 administration (Figure 3(E)), suggesting the markedly protective effect of FG-4592.

### The inflammation was suppressed by FG-4592 in hypoxia-induced renal tubular cell injury

**Figure 4.**
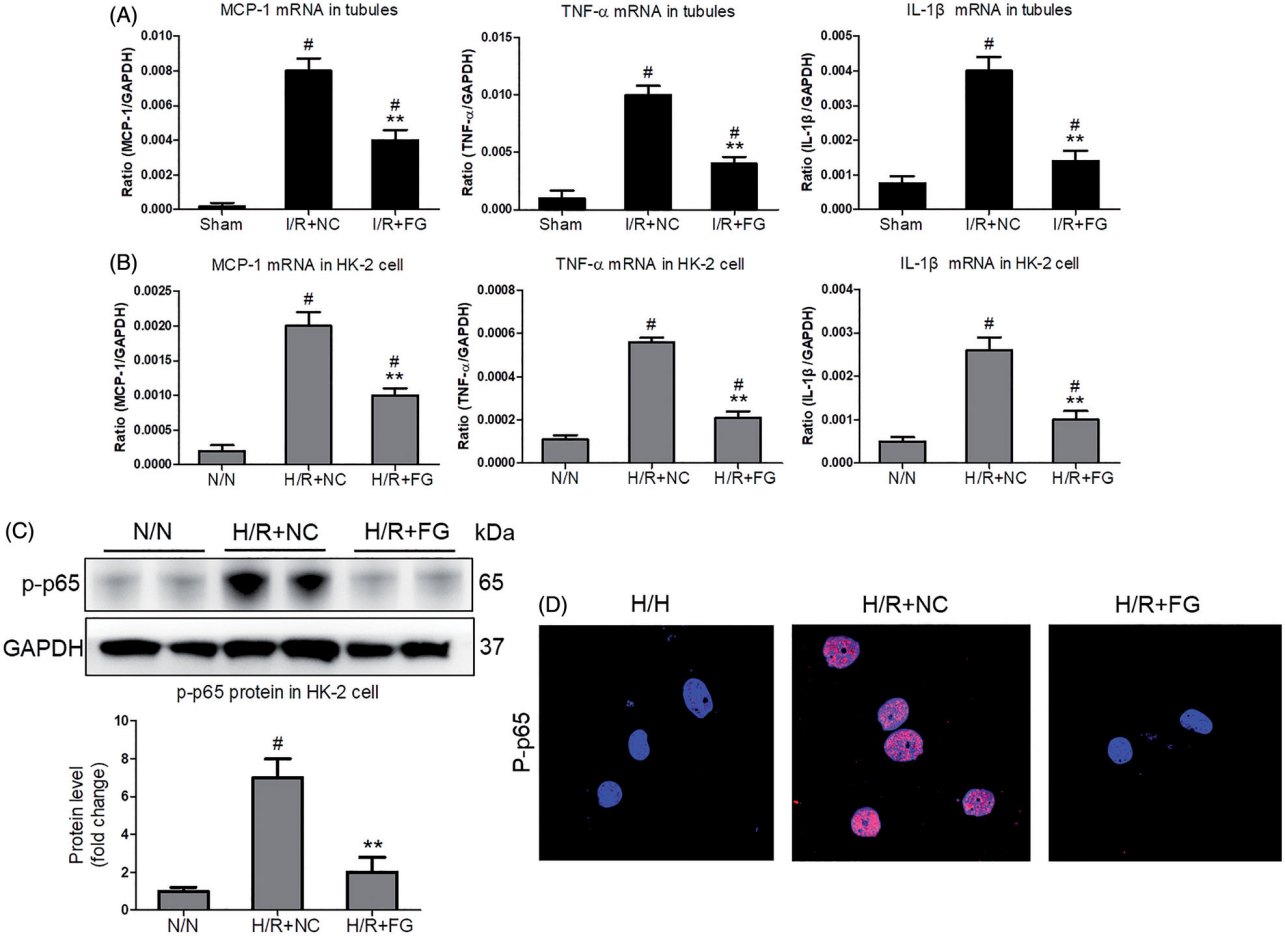
The inflammation was suppressed by FG-4592 in hypoxia-induced TECs. (A) qRT-PCR analysis of cytokine (TNF-α, IL-1β and CCL-2) mRNA expression levels in TECs lysates (*n* = 6). (B) qRT-PCR analysis of cytokine (TNF-α, IL-1β and CCL-2) mRNA expression levels in HK-2 cells. (C) Western blotting analysis of p-p65 protein in HK-2 cells. Quantitative analysis of the Western blots of p-p65. GAPDH was used as the loading control. (D) Representative immunofluorescence staining of p-p65 (×1000). HK-2 were pretreated with or without FG-4592, then cultured with H/R. GAPDH was used as the loading control. All experiments were duplicated three times. **p < .01 vs. I/RI + NC or H/R + NC group, #*p* < .05 vs. I/RI + NC or N/N group, ANOVA followed by Bonferroni correction.

Finally, the inflammation of injury TEC was investigated *in vivo* and *in vitro*. The TECs were isolated from FG-4592 or vehicle-treated mice. Interestingly, the mRNA expression of MCP-1, TNF-α, and IL-1β in FG-4592-treated mice were significantly decreased (Figure 4(A)). Meanwhile, we found that FG-4592 could inhibit H/R-induced mRNA expression of MCP-1, TNF-α, and IL-1β in HK-2 cells (Figure 4(B)). Moreover, suppression of NF-κB p-p65 expression was also found in HK-2 cells with FG-4592 administration (Figure 4(C)). Immunofluorescence staining further confirmed that NF-κB p-p65 expression was decreased in HK-2 cells with FG-4592 pretreatment (Figure 4(D)). These results demonstrated a role of FG-4592 in antagonizing H/R-induced renal tubular cell injury by inhibiting inflammation.

## Discussion

Ischemia/hypoxia-induced AKI is a worldwide public health problem. To date, no effective therapies are available for treatment. FG-4592 is a novel HIF stabilizer that is currently applied for treatment of renal anemia. In this present study, whether FG-4592 can protect against kidney injury *via* inhibiting inflammation is explored. Our results demonstrated that FG-4592 remarkably ameliorated the I/R-induced kidney injury. Reduced TECs injury and consequent inflammatory responses might be the potential mechanisms for the protective effect of FG-4592 on AKI. Thus, our findings definitely offered a clinical potential approach in treating AKI *via* antagonizing the inflammation.

Ischemia/hypoxia is considered the main pathophysiological factor that leads to the development of AKI [14]. Packed with mitochondria and dependent on oxidative phosphorylation, the TECs are particularly vulnerable to ischemic/hypoxic insults, contributing significantly to the loss of kidney function [15]. Accumulating evidence demonstrated that the tubules are not only a victim of injury but also a driving force in the progression of kidney diseases [16]. Moreover, tubule injury alone is sufficient to lead to the progression of kidney disease or fibrosis [17]. Therefore, promoting the repair of injured tubular cells can be the main strategy for the treatment of AKI.

It is well known that activation of the HIF is the central cellular mechanism of adaptation to ischemia/hypoxia. In response to hypoxia, HIF is expressed predominantly in the tubules and works as a master regulator of hypoxic stress [18]. Previous studies have demonstrated that HIF activation protects against I/R- and cisplatin-induced kidney injury, as evidenced by genetic or pharmacological modulation in animal models [10,19–21]. Thus, HIF stabilization using specific small molecule inhibitor is a novel strategy for treatment of AKI. Jamadarkhana et al. [22] found that treatment with TRC160334 (a novel HIF stabilizer) before onset of ischemia or treatment after the reperfusion of kidneys both ameliorated ischemia-induced AKI. In the present study, the effect of FG-4592, which is a novel, orally active, small-molecule HIF stabilizer and is currently in phase IV clinical trial for the treatment of renal anemia, on I/R-induced AKI and the potential mechanism were studied. Interestingly, we found that FG-4592 could ameliorate I/R-induced kidney injury.

More and more evidence indicated that inflammation is the central pathophysiological mechanism of AKI [23]. Moreover, targeting inflammation is considered to be one of the most promising therapies for preventing AKI [24]. Mechanistically, cellular damage and its associated molecular products are thought to be key triggers for inflammation after AKI [25]. Within the kidney, TECs are extremely susceptible to injuries and can release damage–associated molecular patterns, including proinflammatory mediators that promote acute neutrophil– and macrophage–dependent inflammatory responses in AKI [26]. Consistent with previous studies, hypoxic TECs could also facilitate inflammation by promoting inflammatory cell recruitment into the kidney [27], as evidenced by the increased neutrophil and macrophage infiltration. Interestingly, FG-4592 markedly inhibited the interstitial infiltration of macrophages and neutrophils, suggesting the significant inflammation inhibition effect of FG-4592.

Next, the potential regulatory mechanism for the inflammation inhibition effect of FG-4592 was investigated. Given the key role of TECs in the pathogenesis of AKI, we speculated that the protective effect of FG-4592 on AKI was achieved by consequent reduced injuries of TECs. Interestingly, we found that FG-4592 could protect the TECs from hypoxia-induced injury. As for the mechanism, convincing evidence showed that the multiple of genes about metabolism reprogramming was directedly regulated by HIF [28]. Thus, FG-4592 may inhibit inflammation responses by regulating metabolism. Meanwhile, vascular endothelial growth factor, which played a central role in angiogenesis, is also transcriptionally regulated by HIF [29]. Therefore, recovery of oxygen supply through angiogenesis by FG-4592 may also be one of explanation for inflammation inhibition. Furthermore, we also found that the release of inflammatory factors was suppressed by FG-4592 in hypoxia-induced renal tubular cell injury *in vitro* and *in vivo*, suggesting that FG-4592 protects against renal I/R-induced injury by suppressing the release of inflammatory mediator that promotes inflammatory cell recruitment into the kidney.

In summary, in the present study, therapeutic effect of FG-4592 in I/R-induced AKI and the potential mechanism was evaluated. The importance of this investigation is that FG-4592 could be an effective agent for the treatment of AKI in clinic. Diminished TECs injury and suppression of sequence inflammatory responses may be the potential mechanism. Our findings definitely offered a clinical potential approach in treating AKI.

## Supplementary Material

Supplemental Material

## Abbreviations

AKI: acute kidney injury
HIF: hypoxia-inducible factor
GAPDH: glyceraldehyde-3-phosphate dehydrogenase
H/R: ischemia/reoxygenation
HIF-PHI: HIF-PHD inhibitor
I/R: ischemia/reperfusion
IL-1β: interleukin-1β
KIM-1: kidney injury molecular-1
MCP-1: monocyte chemoattractant protein-1
PHD: prolyl hydroxylases
PAS: Periodic Acid-Schiff
TECs: tubular epithelial cells
TNF-α: tumor necrosis factor-α
