# Supplementary material for: Hypoxia-inducible factor prolyl hydroxylase inhibitor roxadustat (FG-4592) protects against renal ischemia/reperfusion injury by inhibiting inflammation
Source: Ren Fail. 2021 May 10;43(1):803–10. doi: 10.1080/0886022X.2021.1915801 (PMC8118507; doi:10.1080/0886022X.2021.1915801)
Supplement: Supplemental Material [file IRNF_A_1915801_SM6850.pdf]

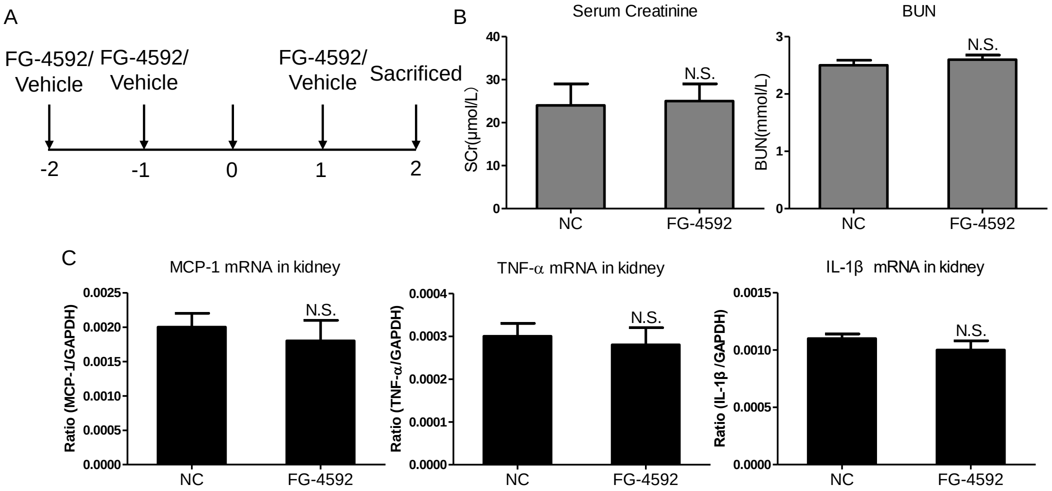

Supplementary Figure 1. The effect of FG-4592 on normal mice. (A) Schematic diagram of the experimental design. In brief, normal mice were administrated with FG-4592 or vehicle. And the mice were sacrificed at 48 h after treatment; (B) Serum creatinine and BUN levels; (C) qRT-PCR analysis of cytokine (TNF-α, IL-β and CCL-2) mRNA expression levels in renal cortex tissue lysates. n=6 mice per group. Data are presented as mean ± SD, N.S., no significance, *T*-test.
